# Protocol for the process evaluation of a counselling intervention designed to educate cancer patients on complementary and integrative health care and promote interprofessional collaboration in this area (the CCC-Integrativ study)

**DOI:** 10.1371/journal.pone.0268091

**Published:** 2022-05-13

**Authors:** Jasmin Bossert, Cornelia Mahler, Ursula Boltenhagen, Anna Kaltenbach, Daniela Froehlich, Joachim Szecsenyi, Michel Wensing, Stefanie Joos, Nadja Klafke

**Affiliations:** 1 Department of General Practice and Health Services Research, University Hospital Heidelberg, Heidelberg, Germany; 2 Department of Nursing Science, University Hospital Tuebingen, Tuebingen, Germany; 3 Institute for General Practice and Interprofessional Care, University Hospital Tuebingen, Tuebingen, Germany; UNITED KINGDOM

## Abstract

**Background:**

Conducting a process evaluation is essential to understand how health interventions work in different healthcare settings. Particularly in the case of complex interventions, it is important to find out whether the intervention could be carried out as planned and which factors had a beneficial or hindering effect on its implementation. The aim of this study is to present the detailed protocol of the process evaluation embedded in the controlled implementation study CCC-Integrativ aiming to implement an interprofessional counselling program for cancer patients on complementary and integrative health care (CIH).

**Methods:**

This mixed methods study will draw upon the “Consolidated Framework for Implementation Research” (CFIR) combined with the concept of “intervention fidelity” to evaluate the quality of the interprofessional counselling sessions, to explore the perspective of the directly and indirectly involved healthcare staff, as well as to analyze the perceptions and experiences of the patients. The qualitative evaluation phase consists of analyzing audio-recorded counselling sessions, as well as individual and group interviews with the involved persons. The quantitative evaluation phase applies questionnaires which are distributed before (T0), at the beginning (T1), in the middle (T2) and at the end (T3) of the intervention delivery.

**Discussion:**

This protocol provides an example of how a process evaluation can be conducted parallel to a main study investigating and implementing a complex intervention. The results of this mixed methods research will make it possible to identify strengths and weaknesses of the team-based intervention, and to target more specifically the key factors and structures required to implement healthcare structures to meet patients’ unmet needs in the context of CIH. To our knowledge, this study is the first applying the CFIR framework in the context of interprofessional CIH counselling, and its results are expected to provide comprehensive and multidisciplinary management of cancer patients with complex supportive healthcare needs.

## Background

Cancer patients’ health-related quality of life (HRQoL) is most negatively affected from the time of cancer diagnosis until the end of chemotherapy. In this demanding phase, many patients seek help and orient themselves towards complementary and integrative health care (CIH) [1]. This is reflected by the fact that up to 80 percent of all cancer patients would like to see greater consideration given to complementary naturopathic approaches [2]. For some CIH procedures such as Yoga, Qi Gong, acupuncture, individual herbal medicines, and aromatherapy, positive effects regarding quality of life and various clinical parameters, such as the reduction of neuropathic symptoms [3], have scientifically proven benefits [4, 5]. However, CIH approaches also entail risks. For example, there are interactions between chemotherapeutic drugs and herbal medicines or dietary supplements, which up to now receive little attention in clinical practice [6]. In addition, patients often make use of services outside the conventional healthcare (e.g. alternative practitioners) with unknown health and financial consequences. Since cancer patients do not inform their treating physicians about this in more than 50% of cases, this poses a threat to the patients’ health, physician-patient relationship, and might lead to a discontinuation of conventional therapy [7, 8].

Given this situation, the study CCC-Integrativ developed an evidence-based interprofessional CIH counselling program that incorporates workforce diversity and provides patient-, provider-, and system-level interventions [9, 10]. The aim of the study is to empower and inform patients in the first six months after diagnosis (including relapse or progress) by providing individual advice (3 consultation sessions within 3 months) on the opportunities and risks of CIH, to enable them to make informed decisions on whether and which CIH they wish to use. The outcome evaluation study of the study CCC-Integrativ has been designed as a naturalistically controlled, non-randomized study with target parameters at the patient, provider and system level, whereby the leading confirmatory hypothesis is at the patient level. The main target parameter is patient activation, measured with the PAM-13 [11], which is collected at baseline (T1), after 3 months (T2) and in follow up, six month after baseline (T3). The total duration of the intervention phase is 18 months. For the individual study participant, the study lasts 6 months including a follow-up survey (intervention group and control group). Further information on the study and the outcome evaluation can be found in the protocol of the main study [9].

### CCC-Integrativ intervention

The project aims to implement and evaluate the interprofessional, evidence-based counselling programme for cancer patients interested to receive counselling and education in the field of CIH. In addition, the intervention is intended to promote interprofessional cooperation and cross-sectoral knowledge transfer in this healthcare context [12]. The present project builds on this expanded understanding of CIH counselling, which includes training and consulting for patients where necessary. Consultation follows an evidence-based approach that integrates patient preferences, research evidence, and individual medical and nursing expertise. Relevant competencies of the evidence-based approach for counselling are imparted to the medical and nursing healthcare professionals in a 13-day blended learning program. Thematic aspects of the blended learning program include communication strategies as well as consultation-specific aspects in different situations of an oncological disease. It is expected that improving the above-mentioned competencies will also improve clinical outcomes as well as health-related quality of life, which may lead to lower utilization of health care services in the future [13]. In addition, training on interprofessional collaboration (TEAMc workshops) was conducted for each consulting team at each of their locations.

**Outcomes at patient level**: Patient-level outcomes aim to strengthen patient empowerment, health-related quality of life, address unmet needs, and reduce the use of risky healthcare services, such as alternative healthcare practitioners without professional training.

**Outcomes at provider level**: Provider-level outcomes aim to improve interprofessional cooperation between physicians and nurses and to enhance job satisfaction within the care setting.

**Outcomes at system level**: System-level outcomes aim to improve the appropriate use of health services and to increase knowledge across healthcare sectors in the field of CIH.

### Rationale of the CCC-Integrativ process evaluation

Attached to the outcome’s evaluation is a process evaluation, which is the focus here. The aim of the process evaluation is to provide insight into intervention fidelity, barriers and facilitators of the implementation processes, as well as of healthcare providers’ experiences and patients’ perspectives impacting on the implemented healthcare structures [14]. The relevance of a process evaluation arises from the fact that the implementation of a new intervention in routine care is challenging [15]. The fidelity of an intervention is defined as the degree to which it is implemented as intended [16]. It reflects adherence to the content, frequency, duration and scope of the planned approach [17]. Lower intervention fidelity may reduce the effectiveness of an intervention due to the fact that not all components are available in the required dose. Conversely, the reverse is also possible: adapting interventions may actually contribute to effectiveness because they are better tailored to local needs and conditions [15]. As a result, insights into intervention fidelity and its related factors enables implementation strategies to be tailored to the identified performance gaps and underlying causes [18].

The process evaluation of this study is guided by the central questions “Could the new form of care be implemented as it was planned (intervention fidelity) and what are the key enablers or obstacles from the perspective of patients and providers? To answer this question, three sub-studies are planned that address the following issues:

1. Evaluation of the interprofessional counselling with regard to intervention fidelity and adaption (sub-study 1).
2. Evaluation of barriers and facilitators for implementation as well as the development among teams with regard to interprofessional cooperation (sub-study 2).
3. Evaluation of patient acceptance and experiences regarding interprofessional, evidence-based counselling (sub-study 3).

## Materials and methods

In the following, the methodological procedure of the process evaluation as a whole and of the three sub-studies is described.

### Study setting

The study takes place at the four participating comprehensive cancer centres (CCCs) of excellence for oncology in Baden-Wuerttemberg (Freiburg, Heidelberg, Tuebingen-Stuttgart, Ulm), Germany. The CCCs in Baden-Wuerttemberg belonging to the 14 oncological centers of excellence in Germany and are characterized by comprehensive treatment of cancer patients as well as in cancer research. CCCs must fulfil all criteria of an oncology center. This includes, among other things, defined guideline numbers. According to the medical discipline, these range from 150 to 800 newly admitted cancer patients per year [19].

### Study design

For the process evaluation, a mixed-methods design was chosen to analyze intervention fidelity, barriers and facilitators for implementation as well as healthcare providers’ and patients’ experiences. The process evaluation is divided into three different studies, each consisting of a qualitative and a quantitative part and refers to the intervention group only.

### Sample size

Power calculation was only performed for the primary endpoint (PAM-13) of the main study [9]. Consistent data on the distribution of the primary endpoint PAM-13 is found in the literature. On this basis, a sample size of 669 patients is calculated (type 2 error of 0.10 and ratio of 2:1 between intervention n = 446, and control group, n = 223). Assuming a drop-out of 30%, 638 patients must be included in the intervention group, and 319 must be included in the control group. For pragmatic considerations regarding the implementation character of the study (existing staff, established structures), we aim to recruit 1000 patients in the intervention group and 500 patients in the control group. With a sample size of 1500 patients (1000+500) minus 30% dropouts, a group difference of 3.2 points on the PAM-13 would have a power of 90 [9]. A power calculation for the mixed-methods process evaluation was not necessary.

### Patients eligibility criteria

Patients whose data are used for the process evaluation are oncology patients in the four participating CCCs of the intervention group. A detailed description of the inclusion and exclusion criteria can be found in the study protocol of the main study [9].

### Study population and sampling

**Patients.** Recruitment of the intervention group has started in the first quarter of 2021. Patients are made aware of the project by physicians and nurses, as well as by flyers and patient information days. At the time of initial contact, the study nurse will provide information about the study CCC-Integrativ and will send written study information, informed consent and information on data protection in the event of willingness to participate in the main study and its process evaluation.

**Healthcare providers.** The sample of healthcare providers is divided into directly and indirectly involved providers. "Directly involved" includes those who are part of the interprofessional consultation tandem of the respective CCCs (physicians n = 2–3, nurses n = 2–3 per CCC). "Indirectly involved" includes other CCC staff not conducting CIH patient counselling but working in medical and nursing management positions (physicians n = 1–2, nurses n = 1–2 per CCC). Participation is voluntary for all providers.

### Theoretical frameworks for the process evaluation

Two frameworks were used for designing this process evaluation. 1) The concept of fidelity refers to study 1 [20]. Here the focus is on ’intervention fidelity’ to be able to conduct a quality assessment to proof whether the intervention was implemented as planned or adapted.

2) The Consolidated Framework for Implementation Research (CFIR) was applied to analyze the enablers and barriers of the implementation processes. The CFIR is an established implementation framework and comprises 39 constructs organized across five main domains on which the present process evaluation is also oriented [14].

1. *Intervention characteristics*: Intervention aspects that may affect implementation success, including its perceived internal or external origin, quality and strength of evidence, comparative advantage, adaptability, testability, complexity, quality and presentation of design, and cost [14, 21].
2. *Outer setting*: External influences on the intervention, including the needs and resources of patients, the openness to the outside environment or the level at which the implementing organization is networked with other organizations, peer pressure and external policies and incentive [14, 21].
3. *Inner setting*: Attributes of the implementing organization such as team culture, compatibility and relative priority of the intervention, structures for setting objectives and feedback, leadership commitment and the climate for implementation [14, 21].
4. *Characteristics of individuals*: Convictions, knowledge, self-efficacy and personal characteristics of the individual that can influence the implementation [14, 21].
5. *Process of implementation*: Phases of implementation such as planning, implementation, reflection and evaluation, and the presence of key stakeholders and influencers of the intervention, including opinion leaders, stakeholder engagement and project advocates [14, 21].

### Plan and purpose of the CCC-Integrativ study’s process evaluation

In Table 1, a summary of the 3 studies is shown with regard to the focus and the data sources for the process evaluation. S2 Fig provides a timeline of the data collection and analysis of the planned process evaluation.

**Table 1:** Overview of the three studies of process evaluation.

	Evaluation focus	Quantitative date source	Qualitative date source
**Study 1**	• Intervention fidelity	• Self-developed questionnaire with 5 Likert scales for the initial (5 items) and follow-up (6 items) counselling	• Audio recordings of several consultation sessions
**Study 2**	• Evaluation of the healthcare providers’ perspective (including barriers and facilitators)	• Interprofessional Socialization and Appreciation (ISVS) • Interprofessional Cooperation (AITCS-II) • Job Satisfaction (WCW) • Competencies • Online questionnaire for evaluating the blended learning program	• Focus groups (location-specific and cross-location) • Individual interviews
**Study 3**	• Experiences and perceptions of the patients	• Self-developed questionnaire for patients (initial and follow-up consultation)	• Individual interviews

#### Study 1: Fidelity of the interprofessional CIH counselling

The evaluation of the interprofessional counselling focuses on the implementation fidelity as described in the section “CCC-Integrativ Intervention”. In order to address the question of fidelity in detail, study 1 aims to answer the following questions:

- How could the communication goals (interprofessional, evidence-based, patient-centered, activating) according to the study’s blended-learning training program be achieved?
- What are the main contents of the interprofessional consultation?
- Was the counselling adapted to local settings, and how?
- What influence did the format (online or face-to-face) have on the counselling?

#### Data collection and data analysis

*Quantitative data*. Within the quantitative part of study 1, the interprofessional counselling will be evaluated from the healthcare providers and patients’ point of view using a self-developed questionnaire with 5 Likert scales for the initial (5 items) and follow-up (6 items) counselling. The questionnaires used within this study were developed according to the evaluation of a psychological consultation program by Walther H [22] and some items were author-developed. Measured aspects in the context of the patient survey are questions about the CIH consultation sessions as well as the consultation atmosphere. In addition, the items 4, 10, 16 (subscale 3) of the validated questionnaire ’Patient Satisfaction with Cancer Treatment Education (PS-CaTE)’ [23] were adopted for the patient questionnaire of the initial and follow-up survey. These questions aim to determine whether patients were satisfactorily informed about complementary therapies, if enough time was available for questions, and if the information was sufficient to make decisions about the use of complementary therapies [23]. Within the context of the healthcare providers survey, the focus is also on the added value of interprofessional consulting as well as satisfaction with one’s own consulting performance. In order to ensure that all questions are understandable, the questionnaires will be piloted in advance. Then, a total of 240 counselling per CCC (n = 960 in total) will be evaluated. This corresponds to approximately 60 counselling per consultant (thereof n = 35 initial and n = 25 follow-up counselling). The evaluation of the questionnaires will be carried out consecutively. For each questionnaire, the calculation of a total score and the calculation of the scores of the individual item will be made. Furthermore, a description of the frequencies with 95% CIs pooled, separately by CCC, is planned. Additionally, an analysis will be carried out to show whether the evaluation of physicians and nursing staff correlate with those of patients and what differences there are between the individual groups in terms of the different CCCs, age, professional experience, gender, interprofessional and monoprofessional consultation. Also subject of the analysis is the correlation of the evaluation of the counselling with the main outcome of patient activation as well as with content wise relevant secondary outcomes. The evaluation takes place at three different points in time (consultation month 1, 9)

*Qualitative data*. In addition, qualitative information on the fidelity of the intervention will be collected in order to be able to assess whether the intervention was carried out as planned (intervention fidelity). For this purpose, audio recordings of several consultation sessions at each CCC (n = 4–8) will be recorded. The choice of the qualitative evaluation method used for the audio recordings according to Kuckartz [24] with a complementary focus on patient-oriented communication was determined by conducting a scoping review based on the PRISMA schema [25] (the publication of the scoping review will be following). The structuring qualitative content analysis according to Kuckartz is understood as the inductively developed category formation along texts up to the deductive implementation of categories. In the context of this study, the analysis of the data is based on the content-structuring and type-forming content analysis according to Kuckartz [26]. Furthermore, following the results of the scoping review, the deductive-inductive category system will focus on patient-centered communication as well as on the other CCC-Integrativ communication goals: interprofessional, activating, evidence-based.

#### Study 2: Evaluation of the healthcare providers’ perspective

The objective of study 2 investigates the acceptance as well as barriers and facilitators for the implementation in daily care from the healthcare providers’ perspective and focuses on the following questions:

- How do the healthcare providers from the CCCs experience the introduction of a new integrative oncological care structure?
- What are the challenges and barriers that need to be known and considered for implementation processes?
- How does interprofessional cooperation develop?
- How does cross-sectoral cooperation work?
- To what extent has the blended learning format offered support for healthcare providers in developing their skills regarding consultation competencies?

#### Data collection and data analysis

*Quantitative data*. The quantitative survey of the directly involved healthcare providers includes Interprofessional Socialization and Appreciation (ISVS), Interprofessional Cooperation (AITCS-II), Job Satisfaction (WCW), and questions regarding their competence gain [27, 28] at four time points (T0 baseline, T1 before intervention, T2 midline, T3 end of intervention). Furthermore, an evaluation of the training program (blended-learning) takes place after each of the 13 days of training by means of an anonymous online questionnaire. The blended-learning evaluation addresses the aspects "informative content", "practical relevance", "motivating", and “appropriateness of content”. Another part of the quantitative evaluation is a survey of the primary care providers within the quality circles. During the quality circles offered by the German Association of General Practitioners, the project with specific CIH contents is presented by a moderator and subsequently evaluated by means of a questionnaire by the general practitioner’s association.

*Qualitative data*. In addition to the questionnaires, semi-structured individual interviews with the direct involved consultation teams (physicians n = 2–3 and nurses n = 2–3) and providers from the management level (n = 2–3) are planned for each CCC. Based on comparable studies, it is assumed that theoretical saturation is reached with this number of interviews, meaning that conducting more interviews would not yield any additional insights [29, 30]. The qualitative interviews will focus on acceptance as well as inhibiting or promoting factors for implementation of the interprofessional CIH consultation services in regular care and the perspectives of the various participants over time will be evaluated. In addition, site-specific interprofessional focus groups with the CCC consultation teams are planned at two different time points, three months after the start of the intervention and at the end of the intervention. Once the intervention phase has been completed, there is also an interprofessional focus group planned across the CCC locations. The qualitative guidelines were developed on the basis of a literature search in the preparatory phase and all transcribed data will be evaluated by means of qualitative content analysis according to Mayring [31] in compliance with the Consolidated Criteria for Reporting Qualitive Studies (COREQ) [32]. The procedure is strictly systematic, comprehensible, and reproducible and thus strongly intersubjectively verifiable. Within the approach of content analysis, a number of concrete qualitative content analytic techniques have been differentiated, which are guided by the basic processes of summarization, explication, and structuring. The evaluation in the context of study 2 is based on the summarizing content analysis. Content summary analysis involves paraphrasing the material in order to delete less relevant passages or passages with the same meaning (first reduction). Subsequently, similar paraphrases will be summarized (second reduction). This will reduce the material by statements that overlap at the level of generalizations [33].

#### Study 3: Evaluation of the patients’ perspective

The objective of study 3 aims to evaluate the patient’s point of view regarding the interprofessional CIH counselling and will focus on the following questions:

- How do patients perceive the interprofessional counselling and which subjective benefits do they experience from them?
- Which challenges arise when implementing the recommended counselling content in the everyday life of patients?

#### Data collection and data analysis

*Quantitative data*. The quantitative part of study 3 uses the self-developed questionnaire for patients (initial and follow-up consultation), which was already described in study 1. That means that also in this case, a total of 960 questionnaires will be analyzed using the same evaluation method as in study 1. Patient questionnaires and provider questionnaires will be matched so that analyses can be conducted with regard to a specific CIH consultation.

*Qualitative data*. Within the qualitative approach, 8–10 patient guideline-based individual face-to-face or telephone interviews per CCC (in total *n* = 32–40) will be carried out. The patients will be selected with the help of a sampling matrix aimed at maximum variation [34] and then invited to the interview in order to examine a broad and varied picture of the patients’ perspectives. Depending on the patient’s preferences, the interviews will be conducted on site in the CCCs or by telephone or video conference. As in study 2, the interviews will be also transcribed and analyzed according to Mayring [31] in compliance with the quality criteria of qualitative research (COREQ). This also applies to the direction of the analysis, which will be in line with the summary content analysis as described in the qualitative part of study 2 [33].

Using a mixed-methods approach, three sub-studies are conducted within the framework of the process evaluation in order to address the CFIR domains: intervention characteristics, outer setting, inner setting, characteristics of individuals, and process of implementation. In this context, the triangulation of qualitative and quantitative data enables a valid conclusion to be drawn with regard to the underlying research questions within the process evaluation.

### Ethics and safety considerations

#### Ethics committee

The study will adhere to prevailing guidelines, including the Declaration of Helsinki and the European Data Protection Law. Prior to the start of the study, an ethics application for the study protocol (Version 2, 27 November 2019) for the main controlled study was submitted to the ethics committees of the participating medical faculties (Freiburg, Heidelberg, Tuebingen, Ulm) for review. All participating medical faculties received a positive vote, and Heidelberg received a positive vote for the process evaluation with the identification S-307/2020 on November 24, 2020.

#### Participant privacy and safety

The participation of patients and participants is voluntary. All participants who choose to participate in the study provide written informed consent. The consent can be withdrawn at any time, without giving reasons and without disadvantages for further medical care. All names of participating patients and all other confidential information are subject to medical confidentiality and the provisions of the EU DSGVO of 25.05.2018. Data will only be passed on in pseudonymized form. In the event of withdrawal from the study, any (data) material that has not yet been pseudonymized or has already been pseudonymized will be destroyed.

#### Availability of data and material

The datasets used and/or analyzed after completing the current study will be available from the corresponding author upon reasonable request.

## Discussion

Process evaluations are essential to better understand the context factors and everyday processes and experiences, to know how, why, for whom, and under which circumstances healthcare interventions work best. This paper outlines the protocol and the planned procedure of the CCC-Integrativ process evaluation, which is conducted in addition to the outcome evaluation, across the four CCCs in Baden-Wuerttemberg, Germany.

This protocol provides an example of how to conduct a process evaluation parallel to an outcome’s evaluation within a controlled study. It is expected that these results will help to tailor the interprofessional consultation intervention according to patients’ needs as well as to the providers’ possibilities and preferences. By following the study design described in this paper, it will be possible to identify the central mechanisms of the overall success of the intervention fidelity from different perspectives (i.e., from patients, providers, system and context).

By identifying barriers and facilitators, and reporting and feedbacking about those in a timely manner, the intervention can be adapted and can benefit future implementations. As a result, the CCC intervention may also strengthen interprofessional collaboration and patient-centered communication over the long term. This is supported by current evidence, which states that the exchange of expertise and knowledge between professional groups not only promotes job satisfaction, but also patient centeredness and quality of cancer care [35].

The CFIR framework guided the study design of this process evaluation, as this framework has been very established in implementation research due to its clarity of concepts and rigor analysis [36]. Another advantage of the current protocol is the use of a mixed methods research design [37]. By combining qualitative and quantitative data, the evaluation is intended to provide an authentic and detailed description of the intervention context, which strengthens interpretation with regard to transferability.

By evaluating the process using different outcome measures, the impact of the program can be assessed to broader sense. To complement this, the concept of intervention fidelity was used to ensure the fidelity of intervention delivery. Fidelity is an essential concept that allows researchers to measure the reliability and validity of behavioral interventions to see to what extent essential core components of an intervention have been implemented in practice [20]. In general, incorporating process evaluation into the design of intervention studies from the onset can help strengthen studies [38].

Due to the current pandemic situation, the data collection of the process evaluation needs to be conducted in a different way as actually planned. In the first part of data collection, the qualitative data recruitment of study 2 will be conducted in a digital format, and this might have an impact on the data quality, as in particular in qualitative research it is important to be present and to be able to interact with each other in person. It is expected that the data collection process can switch to a presence format again in the second half of the data collection.

The study also has some limitations. The self-developed questionnaires have been piloted before their application, but, due to the project’s time plan, they cannot be tested for validity and reliability. However, by adding a validated scale (3 items) to one of the questionnaires used, an attempt has been made to enable comparability with other studies. It should also be mentioned that the health professionals participating in this study and in the counselling may not attend all sessions of an educational program and patients may not remember all aspects of the consultation program. To avoid confirmation bias, the persons responsible for the process evaluation are not involved in the implementation of the intervention.

## Supporting information

S1 Table Spirit checklist 2013. (DOC)

S1 Fig Spirit figure 2013. (DOC)

S2 Fig Times of data collection and analysis. (DOCX)

## Abbreviations

CIH: Complementary and integrative health care
CCC: Comprehensive Cancer Centre
CFIR: Consolidated Framework for Implementation Research
HRQoL: Health-related quality of life
COREQ: Consolidated Criteria for Reporting Qualitive Studies
