# Supplementary material for: Protocol for the process evaluation of a counselling intervention designed to educate cancer patients on complementary and integrative health care and promote interprofessional collaboration in this area (the CCC-Integrativ study)
Source: PLoS One. 2022 May 13;17(5):e0268091. doi: 10.1371/journal.pone.0268091 (PMC9106164; doi:10.1371/journal.pone.0268091)
Supplement: S1 Fig — (DOC) [file pone.0268091.s002.doc]

Figure. Example template of recommended content for the schedule of enrolment, interventions, and assessments.*

|  |  | Study period | | | | | | |
| --- | --- | --- | --- | --- | --- | --- | --- | --- |
|  | **Enrolment** | | **Allocation** | Post-allocation | | | | Close-**out** |
| **TIMEPOINT**** | ***-t_1_*** | | **0** | ***t_1_*** | ***t_2_*** | ***t_3_*** | ***March 2023*** | |
| **ENROLMENT:** |  | |  |  |  |  |  | |
| **Eligibility screen** | X | |  |  |  |  |  | |
| **Informed consent** | X | |  |  |  |  |  | |
| **Allocation** |  | | X |  |  |  |  | |
| **INTERVENTIONS:** |  | |  |  |  |  |  | |
| ***[Patient level]*** |  | |  |  |  |  |  | |
| ***[Providers level]*** |  | |  |  |  |  |  | |
| ***[System level]*** |  | |  |  |  |  |  | |
| **ASSESSMENTS:** |  | |  |  |  |  |  | |
| ***Patient activation (patient level)*** |  | |  | x | x | x | X | |
| ***Health status (patient level)*** |  | |  | x | x | x | X | |
| ***Self-efficacy (patient level)*** |  | |  | x | x | x | X | |
| ***Unmet needs (patient level*** |  | |  | x | x | x | X | |
| ***Quality of life (patient level*** |  | |  | x | x | x | X | |
| ***Depression agitation (patient level)*** |  | |  | x | x | x | X | |
| ***Fatique (patient level)*** |  | |  | x | x | x | X | |
| ***Measure your self-consent and well-being (patient level)*** |  | |  | x | x | x | X | |
| ***Single item literacy screener (patient level)*** |  | |  | x | x | x | X | |
| ***Healthcare utilization questionnaire (Patient level*** |  | |  | x | x | x | X | |
| ***Sociodemographic data (patient level)*** |  | |  | x |  |  | X | |
| ***Medical data (patient level)*** |  | |  | x |  |  | X | |
| ***Interprofessional Socialization and Valuing Scale (Provider level)*** |  | |  | x | x | x | X | |
| ***Interprofessional cooperator*** |  | |  | x | x | x | X | |
| ***Interprofessional job satisfaction*** |  | |  | x | x | x | X | |
| ***Competence gain*** |  | |  | x | x | x | X | |
| ***Competence gain (system level)*** |  | |  | x | x | x | X | |
| ***Satisfaction with information (system level)*** |  | |  | x | x | x | X | |

x

*Recommended content can be displayed using various schematic formats. See SPIRIT 2013 Explanation and Elaboration for examples from protocols.

**List specific timepoints in this row.
