# Supplementary material for: Protocol for the process evaluation of a counselling intervention designed to educate cancer patients on complementary and integrative health care and promote interprofessional collaboration in this area (the CCC-Integrativ study)
Source: PLoS One. 2022 May 13;17(5):e0268091. doi: 10.1371/journal.pone.0268091 (PMC9106164; doi:10.1371/journal.pone.0268091)
Supplement: S2 Fig — (DOCX) [file pone.0268091.s003.docx]

**Supplementary file 1**: Times of data collection and analysis

|  | **Data collection** | | | | | | | |  | |
| --- | --- | --- | --- | --- | --- | --- | --- | --- | --- | --- |
|  |  |  | | | | | | | **Data analysis** | |
|  | **Q1-Q4 2020** | **Q1 2021** | **Q2 2021** | **Q3 2021** | **Q4 2021** | **Q1 2022** | **Q2 2022** | **Q3 2022** | **Q4 2022** | **Q1 2023** |
| **Study 1**  **(Focus: Fidelity)**  **● =** Audio recordings  **◊ =** Self-developed questionnaire |  | **◊** | **●** |  | **◊** | **◊** | **●** |  | **●**  **◊** | **●**  **◊** |
| **Study 2**  **(Focus: Health care providers)**  **● =** Interviews, focus group  **◊ =** BL, ISVS, AITCS-II, WCW, Competencies | **◊** | **●**  **◊** |  | **●** | **●**  **◊** |  |  | **●**  **◊** | **●**  **◊** | **●**  **◊** |
| **Study 3**  **(Focus: Patients)**  ● **=** Interviews  **◊ =** Self-developed questionnaire |  | **◊** | **●** | **●** | **◊** | **●**  **◊** |  |  | **●**  **◊** | **●**  **◊** |
